# Integrated network pharmacology and experimental validation to investigate the therapeutic effects and mechanisms of SJZT on hypertensive nephropathy

**DOI:** 10.3389/fphar.2025.1625482

**Published:** 2025-09-24

**Authors:** Bin Chen, Guanghui Wang, Jianbo Zhou, Lina Han, Li Li, Chengbin Sun

**Affiliations:** ^1^ Department of Nephrology, Ningbo Zhenhai People’s Hospital, Ningbo, Zhejiang, China; ^2^ Ningbo University Health Science Center, Ningbo, Zhejiang, China

**Keywords:** SJZT, hypertensive nephropathy, network pharmacology, renal fibroblasts, PPARγ, autophagy

## Abstract

**Introduction:**

Hypertensive nephropathy (HN) is a common complication of hypertension. Clinically, there is an urgent need for new HN treatment strategies. Sijunzitang (SJZT) is widely used in clinical practice, but its therapeutic effects and pharmacological mechanisms in the treatment of HN remain unclear.

**Methods:**

The active components, key targets, and potential pharmacological mechanisms of SJZT in treating HN were investigated through mass spectrometry, network pharmacology, and molecular docking. Subsequently, we validated the therapeutic effects of SJZT and the potential mechanisms using an Angiotensin II (Ang II)-induced HN mouse model and primary renal fibroblasts *in vitro*.

**Results:**

Network pharmacology identified 87 active components and 26 potential therapeutic targets of SJZT in treating HN, among which PPARγ, TNF, CRP, ACE, and HIF-1α were identified as key targets. Molecular docking demonstrated strong binding affinity between the core active components (Licoisoflavone B, Glabrone, and Frutinone A) and PPARγ. Animal experiments revealed that SJZT attenuated renal damage and extracellular matrix deposition in HN model mice. *In vitro* experiments revealed that SJZT suppressed Ang II-induced renal fibroblasts activation, as evidenced by reduced cell viability, α-SMA, and Collagen I expression. Mechanistically, SJZT alleviated hypertensive renal fibrosis through PPARγ upregulation in renal fibroblasts, subsequently inducing autophagy activation.

**Conclusion:**

This preclinical study establishes that SJZT ameliorates HN through a multi-component, multi-target, and multi-pathway mechanism. Key findings confirm that SJZT activates autophagy via PPARγ upregulation, which subsequently inhibits renal fibroblast activation and attenuates HN progression. These results provide a pharmacological foundation for the translational application of SJZT in HN treatment.

## 1 Introduction

Hypertension, a global chronic disease with increasing prevalence (Mills et al., 2020), often leads to hypertensive nephropathy (HN), a major complication progressing to end-stage renal disease (ESRD) (Rossignol et al., 2015; Ruiz-Ortega et al., 2020). HN is now the second leading cause of ESRD after diabetic nephropathy (DN), presenting a significant public health burden (Hong et al., 2025). Currently, dialysis and kidney transplantation remain the only viable treatments for ESRD (Wouk, 2021). However, dialysis impairs quality of life, while kidney transplantation entails inherent risks. Thus, developing novel therapies and clarifying their mechanisms are essential for the HN prevention and management.

Hypertension activates the renin-angiotensin-aldosterone system (RAAS), elevating angiotensin II (Ang II), a key mediator in HN progression (Rucker et al., 2018; Zhang et al., 2021). Ang II drives renal damage by inducing inflammation and fibrosis (Zhang Y. et al., 2023), making it a common tool for HN modeling. Renal fibrosis, characterized by interstitial and glomerular collagen deposition (Li et al., 2025), disrupts parenchyma structure and impairs kidney function, correlating with HN prognosis. Crucially, fibroblast activation and myofibroblast differentiation underlie fibrotic pathogenesis (Yamashita and Kramann, 2024; Zhao et al., 2023), highlighting their inhibition as a therapeutic target for HN.

Traditional Chinese medicine (TCM) offers a promising therapeutic approach for HN due to its favorable safety profile. Sitjunzitang (SJZT), a classical TCM formula first documented in the earliest traditional Chinese medicine formula book “*Tai Ping Hui Min He Ji Ju Fang”*, is composed of four herbs, including *Panax ginseng C.A.Mey., Poria Cocos, Atractylodes Macrocephala Koidz, and Glycyrrhiza uralensis Fisch* (the plant name has been checked with http://www.theplantlist.org) in a ratio of 3:3:3:2, which have been utilized in China and other Asian countries for about one thousand years (Ke et al., 2024). SJZT exhibits spleen-tonifying properties and is clinically used for gastrointestinal disorder (Yang et al., 2025). According to TCM theory, the spleen and kidneys are functionally interconnected, forming the basis for treating renal diseases through spleen modulation (Lv and Bao, 2020). Recent studies demonstrate that the active components of SJZT mitigate kidney damage by inhibiting inflammation, oxidative stress, and fibrosis in chronic kidney disease (CKD) and DN (Chen et al., 2023; Chen et al., 2020; Jia et al., 2024), and ameliorates hypertensive renal damage (Wang et al., 2019). Furthermore, SJZT suppresses inflammation and renal fibrosis in CKD model mice, improving kidney function (Yu et al., 2024). However, the bioactive components and molecular mechanisms of SJZT against HN require further investigation.

For TCM formulas, which contain multiple components and targets, Traditional pharmacological approaches often insufficient for elucidating the complex multi-component and multi-target mechanisms. Network pharmacology, as an emerging interdisciplinary approach integrating computer technology and systems biology to systematically explore the relationships between TCM formulas and diseases (Zhang P. et al., 2023), it creates a comprehensive network that integrates formulas, ingredients, active components, targets, and diseases (Nogales et al., 2022). This approach is highly significant for revealing the pharmacological mechanism of TCM formulas treat diseases based on multiple active components, multiple targets, and multiple pathways (Wang et al., 2024). Molecular docking serves as a powerful tool for predicting ligand-target interactions. providing critical insights into binding affinities and modes essential for drug discovery (Paggi et al., 2024). Thus, network pharmacology and molecular docking play a crucial role in uncovering the complex mechanisms of TCM formulas treatments for diseases, as well as identifying key active components and targets.

In this study, network pharmacology was adopted to identify the active components and targets of SJZT in treating HN, to screen core active components and key targets, and to elucidate the dominant enrichment functions and signaling pathways. Molecular docking was utilized to reveal the binding affinities between core active components and key targets. Moreover, both *in vivo* and *in vitro* experiments were carried out to further validate the therapeutic effects and molecular mechanisms of SJZT on HN, providing evidence for SJZT in the treatment of HN.

## 2 Materials and methods

### 2.1 Network pharmacology and molecular docking

#### 2.1.1 Active components of SJZT and targets prediction

The components of SJZT were retrieved from the Traditional Chinese Medicine Systems Pharmacology Database and Analysis Platform (TCMSP) (https://www.tcmsp-e.com/index.php) (Ru et al., 2014) using *Reshen*, *Baizhu*, *Fuling*, and *Gancao* as keywords. Active components were evaluated by ADME properties (absorption, distribution, metabolism and excretion), with screening criteria requiring: oral bioavailability (OB) ≥30%, drug likeness (DL) ≥0.18, half-life (HL) >4 (Supplementary Table S2).

The targets of SJZT were obtained through TCMSP, DrugBank (https://go.drugbank.com), STITCH (http://stitch.embl.de/), and SwissTaegetPrediction (https://www.swisstargetprediction.ch)(Daina et al., 2019; Knox et al., 2024; Szklarczyk et al., 2016) (Supplementary Table S3). Target names were standardized using Uniprot (https://www.uniprot.org) (UniProt: the Universal Protein Knowledgebase, 2023).

#### 2.1.2 The targets for HN

The targets for HN were screened through multiple databases, including DrugBank, Therapeutic Target Database (TTD, https://db.idrblab.net/ttd/) (Zhou et al., 2024), GeneCards (https://www.genecards.org) (Stelzer et al., 2011), and DisGeNET (http://www.disgenet.org/) (Piñero et al., 2020). After removing duplicate targets, the HN targets were obtained (Supplementary Table S4).

#### 2.1.3 Drug-targets network construction

The drug-target interaction network was constructed by importing the active components of SJZT and the intersection targets of SJZT active components with HN (Supplementary Table S5) into Cytoscape (http://www.cytoscape.org/). Based on the degree values, the top 10 active components were identified (Table 1).

**TABLE 1:** Top 10 core active components of SJZT in the treatment of HN.

Molecular ID	Active component	Source	OB(%)	DL	HL
MOL000098	quercetin	Glycyrrhizae Radix et Rhizoma	46.43	0.28	14.4
MOL001792	DFV	Glycyrrhizae Radix et Rhizoma	32.76	0.18	17.89
MOL004810	Glyasperin F	Glycyrrhizae Radix et Rhizoma	75.84	0.54	15.64
MOL004856	Gancaonin A	Glycyrrhizae Radix et Rhizoma	51.08	0.4	16.82
MOL005320	arachidonate	Panax Ginseng	45.57	0.2	7.56
MOL004912	Glabrone	Glycyrrhizae Radix et Rhizoma	52.51	0.5	16.09
MOL005321	Frutinone A	Panax Ginseng	65.9	0.34	19.1
MOL004884	Licoisoflavone B	Glycyrrhizae Radix et Rhizoma	38.93	0.55	15.73
MOL004891	Shinpterocarpin	Glycyrrhizae Radix et Rhizoma	80.3	0.73	6.5
MOL000422	kaempferol	Glycyrrhizae Radix et Rhizoma/Panax Ginseng	41.88	0.24	14.74

#### 2.1.4 Protein-protein interaction network (PPI)

In order to determine interactions among the intersection targets, we loaded them into the STRING database (https://string-db.org/) (Szklarczyk et al., 2025) to construct PPI network. After exporting the data, we imported PPI network into Cytoscape for further analysis, focusing on calculating topological parameters and identifying key targets. Topological parameters including closeness centrality, degree centrality, and betweenness centrality were calculated using the CytoNCA plugin. Targets with degree values above the median were selected for subsequent analysis (Table 2).

**TABLE 2:** The key targets of SJZT in the treatment of HN.

Gene	Target	Degree	UniProt ID
TNF	TNF	16	P01375
CRP	CRP	15	P02741
ACE	ACE	15	P12821
HIF1A	HIF-1α	14	Q16665
NFKB1	NF-κB	13	P19838
PPARG	PPARγ	13	P37231
AGTR1	AGTR1	12	P30556
MPO	MPO	11	P05164
CASP3	Caspase 3	10	P42574
F3	F3	9	P13726

#### 2.1.5 Gene ontology (GO) and kyoto encyclopedia of genes and genomes (KEGG)

The intersection targets of SJZT active components and HN were subjected to Gene Ontology (GO) and Kyoto Encyclopedia of Genes and Genomes (KEGG) enrichment analysis (conducted using the clusterProfiler package in R), as detailed in Supplementary Tables S6, S7). The standard for selecting significantly enriched functions and pathways was set as FDR <0.05.

#### 2.1.6 Molecular docking

The crystal structures of PPARγ were retrieved from the Protein Data Bank (PDB) database (https://www.rcsb.org/). PyMol was employed to remove water molecules and heterogeneous molecules, and loops were incorporated. The PDBQT files of the PPARγ crystal structures and top 10 active components were generated using AutoDock tools. AutoDock Vina software was employed to perform molecular docking and calculating binding energies. Protein molecules spontaneously bind to small molecules when their binding energy is less than 0 kcal/mol; favorable binding activity is indicated when it is less than −5.0 kcal/mol; and considerable binding activity is indicated when it is less than −7.0 kcal/mol (Liu et al., 2021). Finally, the docking model pose with the lowest binding energy was visualized using PyMol (Supplementary Table S8).

### 2.2 Experimental verification

#### 2.2.1 Preparation method of SJZT

SJZT is composed of *P. ginseng C.A.Mey*. (Liaoning Chaohong Ginseng Industry Co., Ltd., 20230407, Jilin, CN), *Atractylodes macrocephala Koidz* (Quzhou Nankong Traditional Chinese Medicine Co., Ltd., 2307019, Bozhou, CN), *Poria cocos* (Quzhou Nankong Traditional Chinese Medicine Co., Ltd., 23031001, Yunnan, CN), and *G. uralensis Fisch* (Quzhou Nankong Traditional Chinese Medicine Co., Ltd., 20230909, Xinjiang, CN). The preparation method is as follows: Soak the above herbs in 10 times their weight of pure water for 1 h. Boil the mixture and maintain a gentle simmer for 40 min. Collect the decoction liquid by filtering through gauze. Add 8 times the herb weight of pure water to the residue and boil again for 30 min. The liquid is filtered and combined it with the first decoction. Filter the combined extracts through sterile gauze, then concentrate the supernatant to a final volume of 1 L using a rotary evaporator. Freeze the concentrated liquid overnight at −80 °C, followed by lyophilization in a freeze-dryer to obtain freeze-dried powder.

#### 2.2.2 Characterization of the main ingredients in SJZT

To characterize the main ingredients in SJZT, the metabolites were extracted from SJZT first by centrifuging at 12000 rpm (RCF = 13800 × g, R = 8.6 cm) for 15 min at 4 °C. Then, 300 μL of the supernatant was mixed with 1,000 μL of extraction solution (MeOH: ACN: H_2_O, 2: 2: 1, v/v/v) containing deuterated internal standards. The mixture was vortexed for 30 s, sonicated for 5 min in a 4 °C water bath, and incubated for 1 h at −20 °C to precipitate proteins. After another centrifugation step under the same conditions (12000 rpm, 15 min, 4 °C), the supernatant was filtered through a 0.22 μm membrane and transferred to fresh glass vials for LC-MS/MS analysis. A quality control (QC) sample was prepared by pooling equal aliquots of all supernatants. The analysis was performed using a UHPLC system (Vanquish, Thermo Fisher Scientific) equipped with a Phenomenex Kinetex C18 column (2.1 mm × 100 mm, 2.6 μm) and coupled to an Orbitrap Exploris 120 mass spectrometer (Thermo). The mobile phase consisted of 0.01% acetic acid in water (A) and isopropanol: acetonitrile (1:1, v/v) (B). The auto-sampler was maintained at 4 °C, with an injection volume of 2 μL. The mass spectrometer acquired data in data-dependent acquisition (DDA) mode, controlled by Xcalibur software, with ESI source conditions set as follows: sheath gas flow rate at 50 Arb, auxiliary gas flow rate at 15 Arb, capillary temperature at 320 °C, full MS resolution at 60,000, MS/MS resolution at 15,000, stepped NCE collision energy at 20/30/40, and spray voltage at 3.8 kV (positive) or −3.4 kV (negative). Raw data were converted to mzXML format using ProteoWizard (v3.0.24054). Metabolite identification was subsequently performed using the KEGG, PubChem, HMDB, and Herb databases.

#### 2.2.3 Ang II-induced HN mouse model

Twelve-week-old female BALB/c mice were randomly assigned to four experimental groups (n = 5 per group), designated as Control, Ang II, Ang II + Low SJZT, and Ang II + High SJZT. The experimental protocols involving animals in this study were reviewed and approved by the Animal Ethics Committee of Ningbo Zhenhai People’s Hospital (Ethics number: 202310115). After the mice were fed for 1 week, mice were anesthetized with 3% isoflurane, and then the Ang II osmotic pump (1,500 ng/kg/min) or normal saline osmotic pump was implanted under the back skin of mice, the dose of Ang II selected on this study was based on previously published articles (Xia et al., 2013). The Low SJZT group was administered SJZT via oral gavage at a dose of 7.8 g/kg per day, and the High SJZT group received 15.6 g/kg via the same method. The mice were kept for 28 days. Systolic, diastolic, and mean blood pressures in awake mice were monitored weekly using the Softron Biotechnology BP-2010 A non-invasive tail-cuff system (Beijing, CN). Urine was collected for 24 h on day 27–28, and serum and kidney tissue were collected on day 28 for further analysis.

#### 2.2.4 Biochemical measurements

Urine samples were collected after the mice were housed in metabolic cages for 24 h. Blood samples were obtained from the inner canthus artery. Blood urea nitrogen (BUN, mlbio, ml076479, Shanghai, CN), serum creatinine (Scr, mlbio, ml037726, Shanghai, CN), and urinary protein (Nanjing Jiancheng Bioengineering Institute, C035-2-1, CN) quantity were measured on a biochemistry autoanalyzer using commercial kits.

#### 2.2.5 Masson staining

Paraffin tissue sections were dewaxed by xylene, hydrated by gradient ethanol, nucleus was stain Weigert iron hematoxylin, and stained with Masson three-color dyeing kit (Solarbio, G1340, Wuhan, CN). The brief steps are as follow: differentiation with 1% hydrochloric acid alcohol, rinsing with running water, Lichun red acid fuchsin staining for 3 min, and then rinsing with running water, differentiation with 1% phosphomolybdate solution, drying and re-staining with aniline blue solution, washing with 1% glacial acetic acid, dehydration with 95% alcohol and anhydrous ethanol, making transparent with xylene, air drying, sealed with neutral gum, and examined under a microscopic (Olympus, BX53, Tokyo, Japan).

#### 2.2.6 Preparation of SJZT drug-containing serum

Specific pathogen-free (SPF) twelve-week-old female C57BL/6J mice were administered SJZT decoction at a dose of 78 g crude drug/kg body weight, which was converted to 10 times the clinical adult dosage. The drug was dissolved in distilled water and administered via oral gavage at a volume of 0.1 mL/10g body weight. The mice were gavaged twice daily for five consecutive administrations. Blood was collected 2 h after the last administration. Blood samples were allowed to clot at room temperature for 1 h, and serum was separated by centrifugation at 3000 *g* for 15 min at 4 °C. The serum was inactivated in a 56 °C water bath for 30 min to deplete complement, followed by filtration through a 0.22 μm sterile filter under a laminar flow hood. The prepared serum was aliquoted and stored at −20 °C for future use.

#### 2.2.7 Cell culture

Mouse kidney fibroblasts (Procell, CP-M069, Wuhan, CN) were cultured with DMEM (Procell, PCK010, Wuhan, CN) supplemented with 10% fetal bovine serum (FBS; Procell, 164210-500) and 1% penicillin-streptomycin solution (Procell, PB180120). Cells were maintained in a humidified incubator at 37 °C with 5% CO_2_. For subculturing, cells were washed twice with phosphate-buffered saline (PBS, pH 7.4) when they reached 80%–90% confluence, digested with 0.25% trypsin-EDTA at 37 °C, and the reaction was stopped by adding complete medium containing FBS. Cells were gently resuspended and passaged at a 1:3 ratio into new culture vessels.

#### 2.2.8 Western blot

Kidney tissue and cell proteins were extracted by RIPA lysis buffer (Beyotime, P0013B, Shanghai, CN) containing Protease Inhibitor Cocktail (EDTA-Free, 100×). BCA protein quantification kit (Beyotime, P0012S, Shanghai, CN) was used to determine the protein concentration. 40 μg protein in each sample were separated by SDS-PAGE. Primary antibodies (α-SMA, Affinity, AF1032; LC3, Affinity, AF5402; p62, Affinity, AF5384; Collagen I, Affinity, AF7001; PPARγ, Affinity, AF6284; GAPDH, Affinity, AF7021) were added, and incubated at 4 °C overnight, HRP-conjugated secondary antibody (goat anti-rabbit secondary antibody, Boster, BA1054) was added and incubated at 25 °C for 2 h, the ECL reagent (Beyotime, P0018, Shanghai, CN) was used, the results were photographed using a chemiluminescence imaging system, protein expression was analyzed using ImageJ software.

#### 2.2.9 CCK-8

Cells were resuspended and seeded into 96-well plates (5 × 10^4^/mL) and cultured 24 h. Then cell was treated with 10 μM Ang II (aladdin, A424039, Shanghai, CN), or with 5%, 10%, 15%, and 20% serum, or with 5%, 10%, 15%, and 20% SJZT drug-containing serum, or with 10 μM chloroquine (CQ, 10 mM CQ in 1 mL DMSO, macklin, C843545, Shanghai, CN), or with 10 μM GW9662 (10 mM GW9662 in 1 mL DMSO, aladdin, G407990, Shanghai, CN), respectively. After 72 h, added 10 μL CCK-8 (MCE, HY-K0301, Shanghai, CN) solution to each well, incubated for 2 h at 37 °C, and then measured the absorbance at 450 nm by microplate reader (Molecular Devices Co.).

#### 2.2.10 GFP-LC3 detection

Mouse kidney fibroblasts were treated for 72 h with 10 μM Ang II, 15% serum, 15% SJZT drug-containing serum, or 10 μM CQ. After the cells were treated, replaced with fresh medium, and added 10 μL AD-Mcherry-GDP-LC3B virus (Beyotime, C3011, Shanghai, CN) to each well. After culture for 24h, fixed with 4% paraformaldehyde, the nucleus was stained with DAPI (Yeasen, 40728ES03, Shanghai, CN). Autophagy flow was observed under confocal laser fluorescence microscope (Olympus, FV3000, Tokyo, Japan). GFP (excitation/emission: 488/510 nm), and DAPI (excitation/emission: 358/461 nm) signals were captured.

### 2.3 Statistical analysis

Statistical analyses were conducted using SPSS 22 software. Data were presented as mean ± standard deviation (SD). Comparisons between two groups were conducted using Student’s t-test, while multiple group comparisons were analyzed via one-way ANOVA followed by the Tukey’s honestly significant difference (HSD) test. BP data were analyzed using a 4 × 4 mixed-design ANOVA (Group × Time). For *post hoc* testing, Tukey’s HSD test was used for pairwise comparisons. A p-value less than 0.05 was considered statistically significant.

## 3 Results

### 3.1 Identification of the characteristic components in SJZT

**FIGURE 1:**
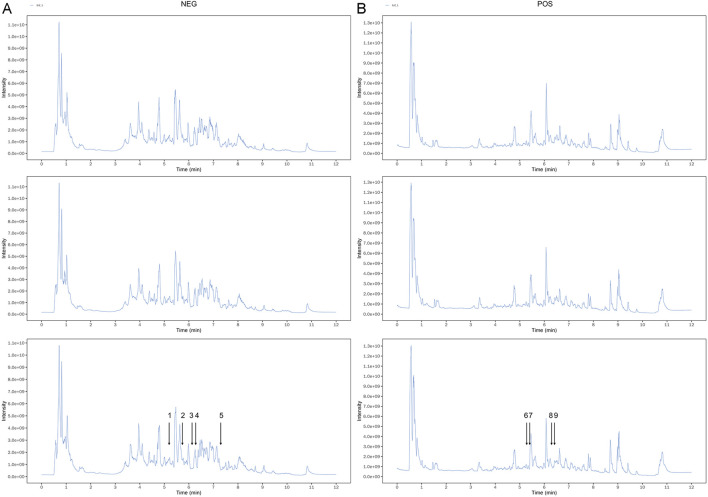
The spectrums of SJZT identified by LC-MS/MS. **(A)** Three batches of SJZT samples analyzed by negative ion mode **(A)** and positive ion mode **(B)**. (1) Isoliquiritin apioside, (2) Quercetin, (3) kaempferol, (4) Vestitol, (5) Poricoic acid A, (6) Ononin, (7) Licochalcone B, (8) Isoformononetin, (9) Ginsenoside Rg6.

To determine the quality of SJZT used in this study, we performed LC-MS/MS analyses on the SJZT using an ultra-high-performance liquid chromatography (UHPLC) system coupled to an Orbitrap Exploris 120 mass spectrometer. Three batches of the decoction were analyzed to enhance the accuracy and reliability of the results. The results demonstrated consistent gradients for each active component across the three batches. The chemical fingerprint of SJZT is presented in Figure 1 and Supplementary Table S1. The retention times of the nine main active components, namely, quercetin kaempferol, poricoic acid A, licochalcone B, isoliquiritin apioside, ononin, ginsenoside Rg6, isoformononetin, and vestitol were 343.9, 367.8, 438.4, 327.8, 313.3, 315.7, 386, 378.2, and 375.5, respectively. Collectively, these data confirm the presence of the main bioactive components in the SJZT, providing robust scientific support for subsequent experiments.

### 3.2 Active components and targets of SJZT

Based on the TCMSP database, we identified 106 active components in SJZT, including 18 from *P. ginseng C.A.Mey.*, 2 from *Atractylodes Macrocephala Koidz*, 11 from *P. cocos*, and 75 from *G. uralensis Fisch*. After removing 1 duplicate active component, 105 unique active components were retained (Supplementary Table S2). Using TCMSP, DrugBank, STITCH, and SwissTargetPrediction, a total of 4859 potential targets associated with SJZT active components were obtained, 1,028 for *P. ginseng C.A.Mey.*, 48 for *Atractylodes Macrocephala Koidz*, 691 for *P. cocos*, and 3092 for *G. uralensis Fisch* (Supplementary Table S3). After removing duplicate targets from different active components, yielded 801 unique targets of SJZT.

### 3.3 HN targets and SJZT active components-targets network

HN targets were screened using DrugBank, TTD, DisGeNET, and GeneCard. After removing duplicate targets from different online databases, 138 unique HN targets were retained (Supplementary Table S4).

**FIGURE 2:**
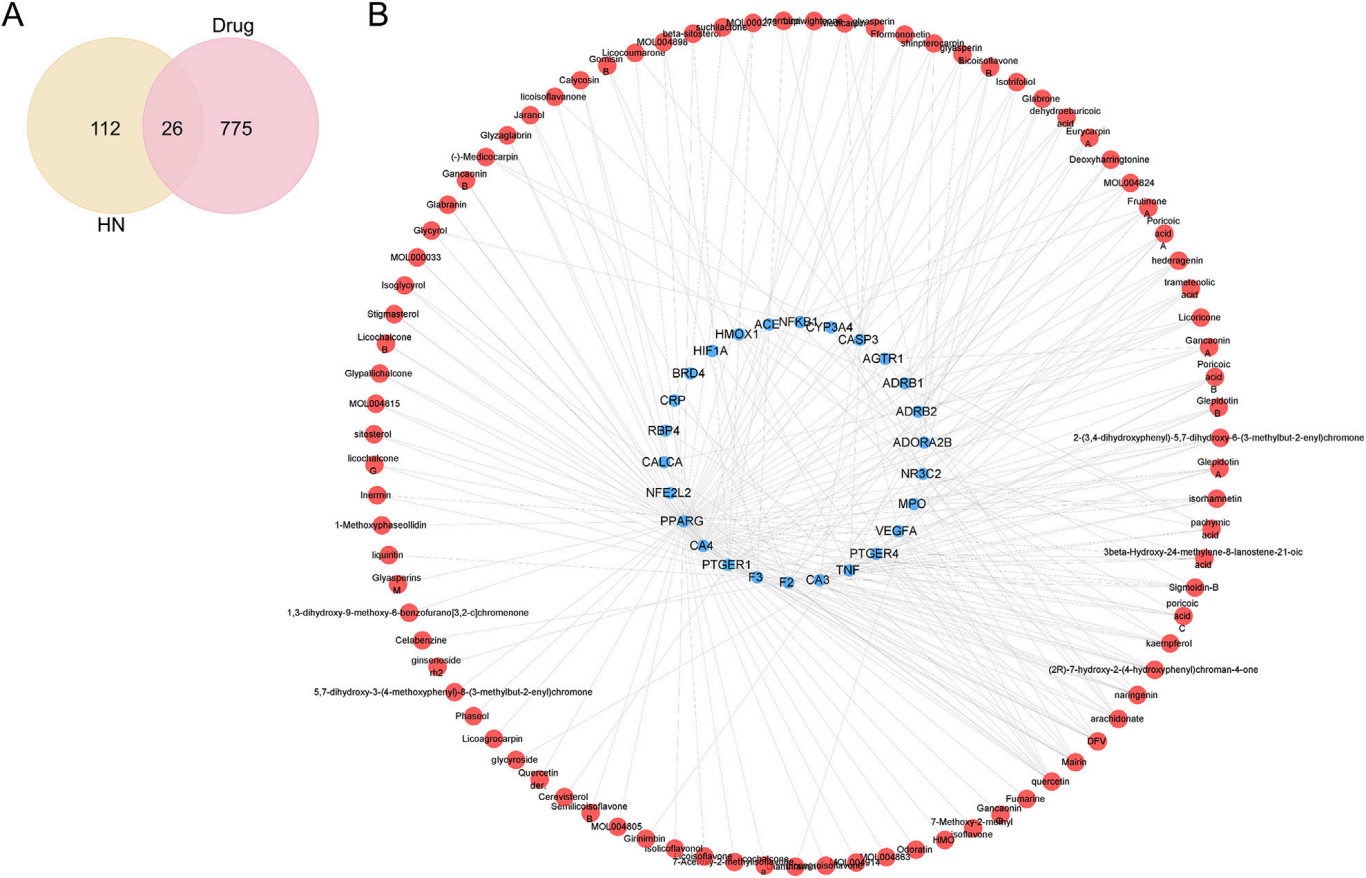
SJZT active components-targets network. **(A)** Venn diagram analysis of the intersection targets between the SJZT active components and HN; **(B)** Construction of the SJZT active components-targets network using Cytoscape. Red represents active components, and blue represents intersection targets.

To identify active components and potential targets of SJZT for HN treatment, we intersected SJZT active components targets with HN targets, yielding 26 intersection targets (Figure 2A; Supplementary Table S5) linked to 87 active components. Next, an active component-target network was constructed via Cytoscape (Figure 2B). Based on closeness centrality, degree centrality, and betweenness centrality values, the top 10 core active components were selected, including quercetin, DFV, Glyasperin F, Gancaonin A, arachidonate, Glabrone, Frutinone A, Licoisoflavone B, Shinpterocarpin, and kaempferol (Table 1). These core active components may play key role in SJZT-mediated HN treatment.

### 3.4 PPI network and the key targets

**FIGURE 3:**
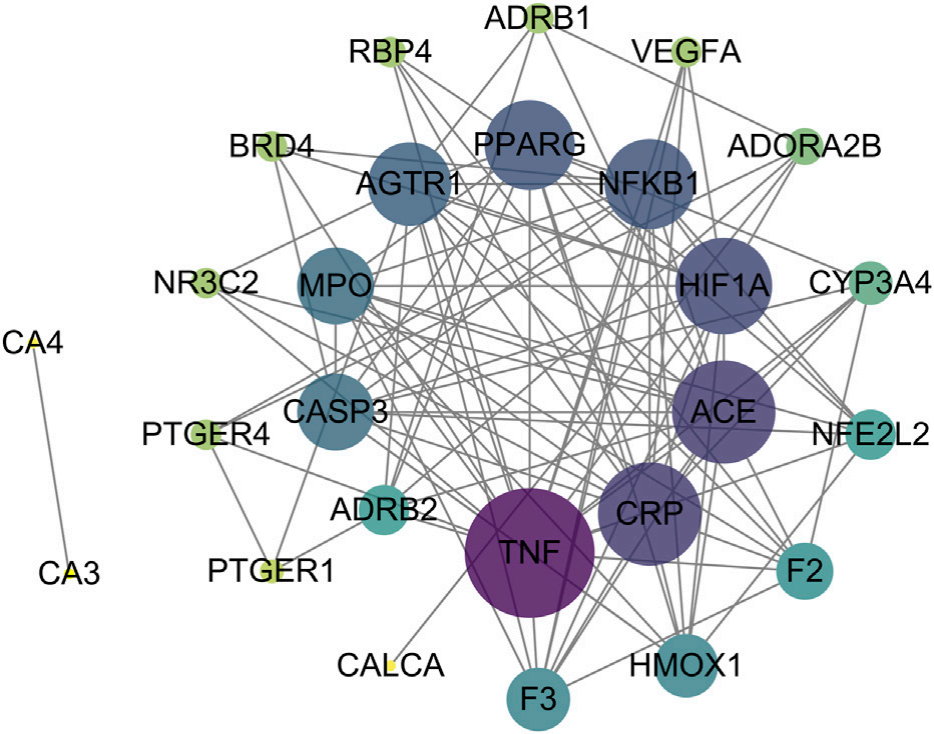
Construction of the PPI network of the intersection targets and screening of the key targets, as the degree value increases, the circle becomes purplier and its area larger, conversely, a decrease in degree value results in a greener color and a smaller size.

To analyze interactions among intersection targets and identify key targets, we constructed a PPI network of the intersection targets using STING database. The network revealed multiple interactions between targets (Figure 3). The data were imported into Cytoscape for topological analysis. Targets were ranked by closeness centrality, degree centrality, and betweenness centrality values, and those exceeding the average for each parameter were selected as key targets, yielding 10 candidates, including PPARγ, TNF, CRP, ACE, HIF-1α, NF-κB, AGTR1, MPO, caspase 3 and F3 (Table 2).

### 3.5 GO enrichment analysis and KEGG pathway analysis

**FIGURE 4:**
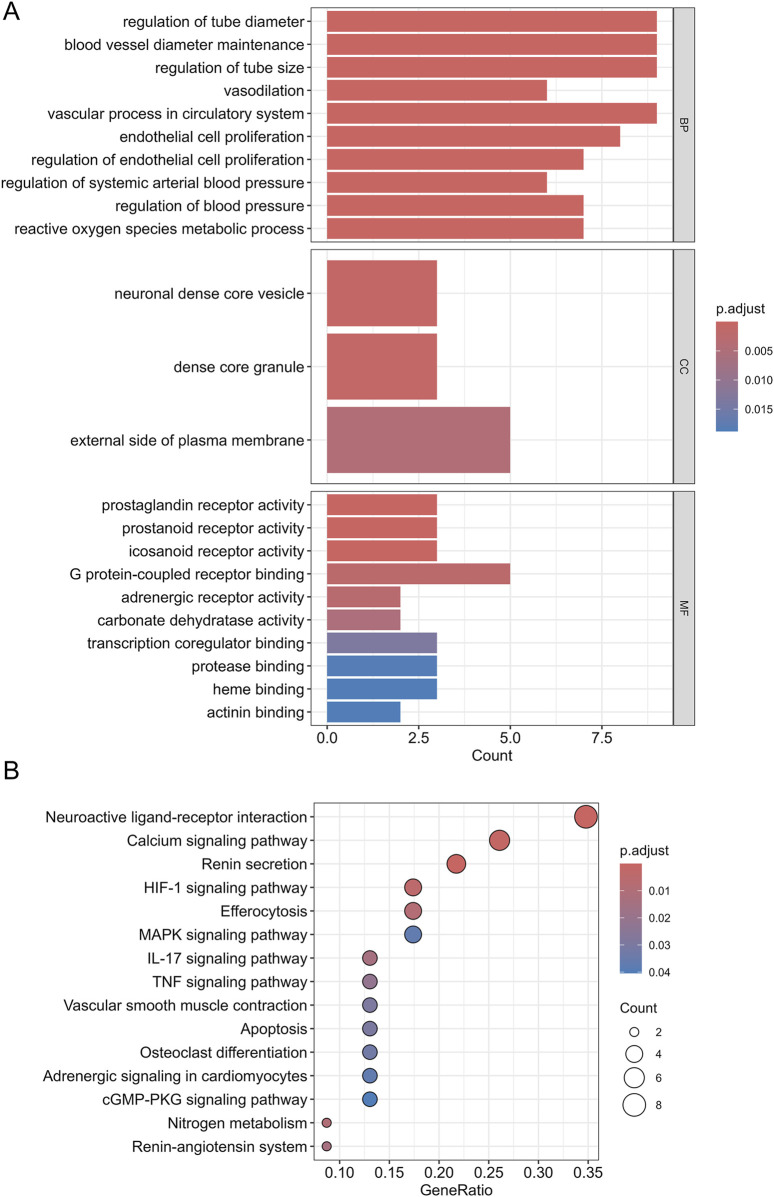
GO functional enrichment analysis and KEGG pathway analysis. **(A)** GO enrichment entries for BP, CC, and MF; **(B)** The significantly KEGG pathways.

To investigate the primary biological functions of SJZT in HN treatment, we conducted GO enrichment analysis on the 26 intersection targets. The targets were enriched in 23 GO terms (Supplementary Table S6), including 10 biological processes (BP), 3 cellular components (CC), and 10 molecular functions (MF) (Figure 4A). BP analysis indicated that these targets were mainly associated with regulation of tube diameter, regulation of endothelial cell proliferation, regulation of blood pressure, etc.; CC analysis showed that these targets were predominantly located in dense core granule, external side of plasma membrane, etc.; MF analysis indicated that these targets were predominantly involved in carbonate dehydratase activity, prostaglandin receptor activity, G protein-coupled receptor binding, transcription coregulator binding, etc. To reveal the key signal pathways regulated by SJZT in HN treatment, we then performed KEGG pathway analysis, revealing enrichment in 16 pathways (Figure 4B; Supplementary Table S7), including renin secretion, MAPK signaling pathway, calcium signaling pathway, renin-angiotensin system (RAS), and cGMP-PKG signaling pathway, etc.

### 3.6 Molecular docking

**FIGURE 5:**
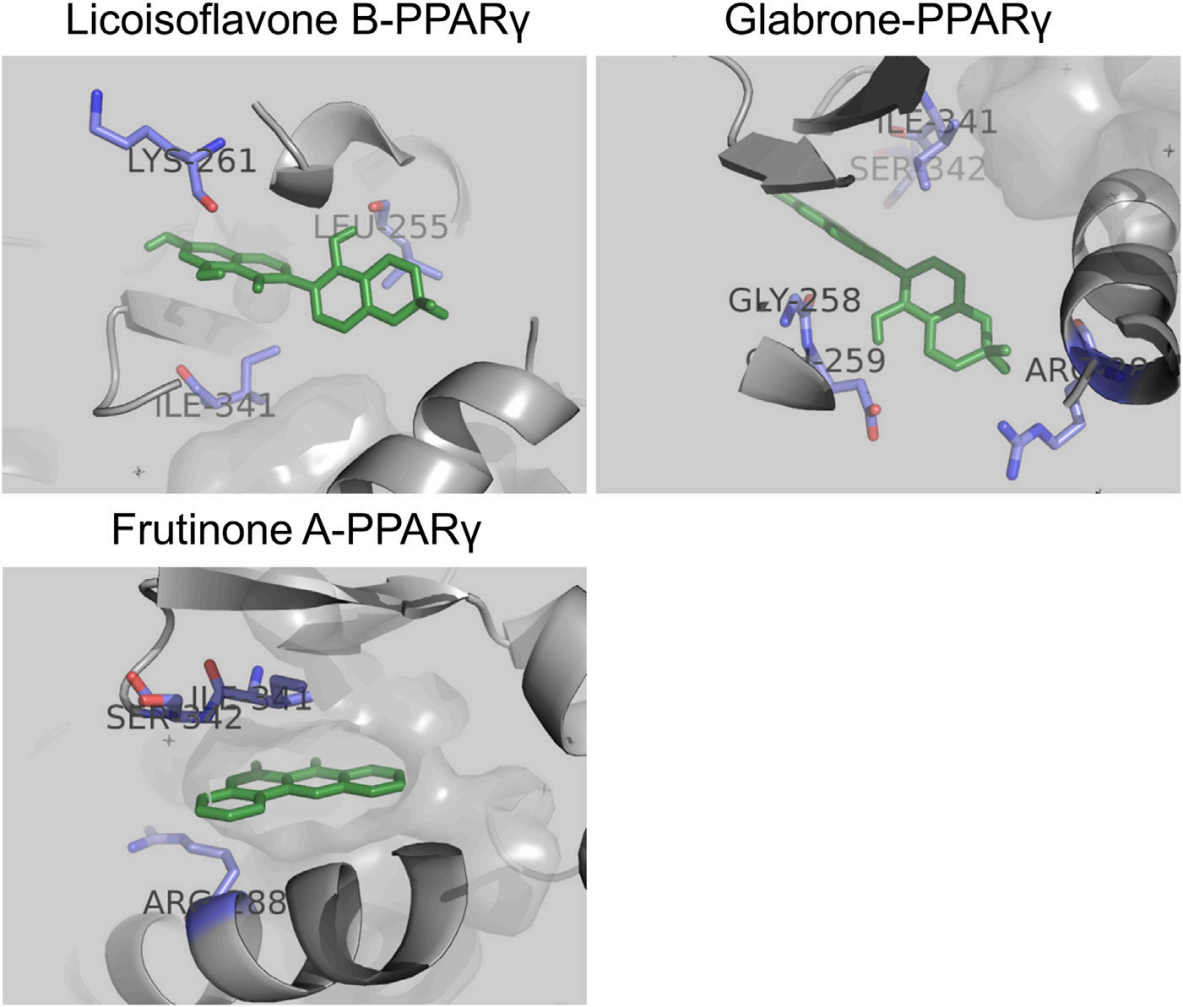
Molecular docking between core active components and PPARγ: Licoisoflavone B-PPARγ, Glabrone-PPARγ, and Frutinone A-PPARγ.

To reveal the binding patterns and affinity between the core active components and key targets, molecular docking analysis was conducted. We select PPARγ for analysis because studies have shown that PPARγ regulates renal fibrosis (Kokeny et al., 2021). The results showed that PPARγ could match 6 out of the top 10 core components (Supplementary Table S8). All components exhibited strong binding activity to PPARγ (protein molecules spontaneously bind to small molecules when their binding energy is less than 0 kcal/mol; favorable binding activity is indicated when it is less than −5.0 kcal/mol; and considerable binding activity is indicated when it is less than −7.0 kcal/mol (Liu et al., 2021)). Among these, Licoisoflavone B (−9.3 kcal/mol), Glabrone (−8.7 kcal/mol), and Frutinone A (−8.6 kcal/mol) showed the lowest binding free energy and the strongest binding activity. Their docking modes with PPARγ are shown in Figure 5.

### 3.7 SJZT attenuated renal damage and renal fibrosis in Ang II-induced HN mouse model

**FIGURE 6:**
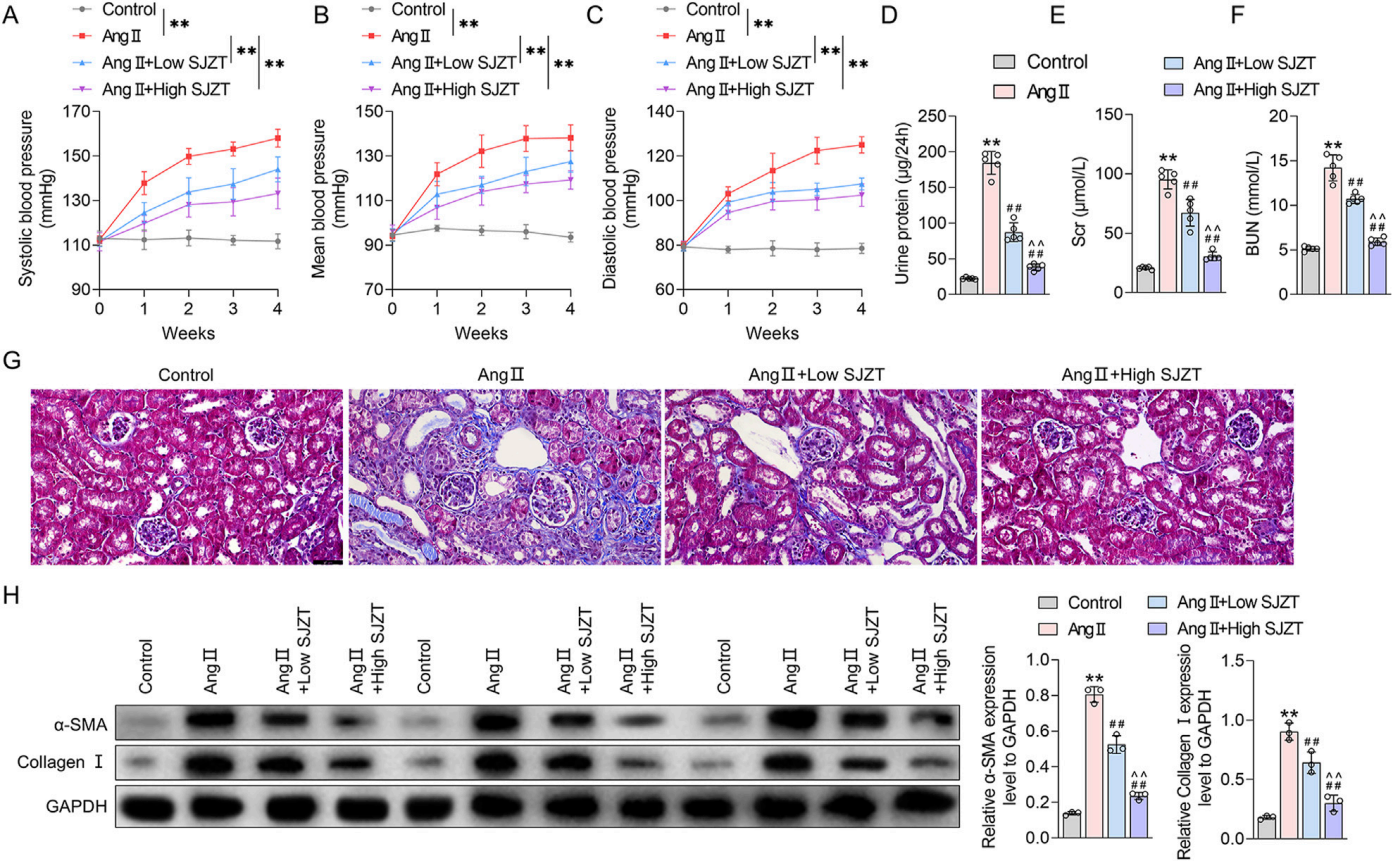
SJZT attenuated renal damage and renal fibrosis in Ang II-induced HN model mice. Systolic blood pressure **(A)**, mean blood pressure **(B)**, and diastolic blood pressure **(C)**. Kits was used to detected 24 h urinary protein **(D)**, Scr **(E)**, and BUN **(F)**; **(G)** Masson staining was used to observe collagen fibers deposition with a scale of 50 μm; **(H)** Western blot was used to detect α-SMA and collagen I expression. **p < 0.01 VS Control, ##p < 0.01 VS Ang II, ^^p < 0.01 VS Ang II + Low SJZT. n = 5.

To evaluate the effect of SJZT on renal fibrosis in HN, a HN mouse model was induced by Ang II infusion and treated with SJZT. We first examined blood pressure in each group of mice. The results demonstrated no significant differences in baseline blood pressure among groups, including systolic blood pressure (SBP), diastolic blood pressure (DBP), and mean blood pressure (MBP). Mice subjected to Ang II infusion exhibited marked increases in SBP, DBP, and MBP, whereas SLZT significantly attenuated the Ang II-induced rise in these parameters (Figures 6A–C). Additionally, the Ang II induced HN mouse model showed elevated 24 h urinary protein, Scr, and BUN, these parameters were lower in the low-dose SJZT-treated group and markedly lower in the high-dose SJZT-treated group (Figures 6D–F). These findings suggested that SJZT markedly attenuated Ang II-induced renal damage in a dose-dependent manner. Further analysis revealed increased collagen deposition, α-SMA, and collagen I levels in the Ang II-induced HN model, which were lower in the SJZT-treated groups, with almost complete abrogation of the development of renal fibrosis by the high dose of SZJT treatment. (Figures 6G,H).

### 3.8 SJZT inhibits renal fibroblast activation

**FIGURE 7:**
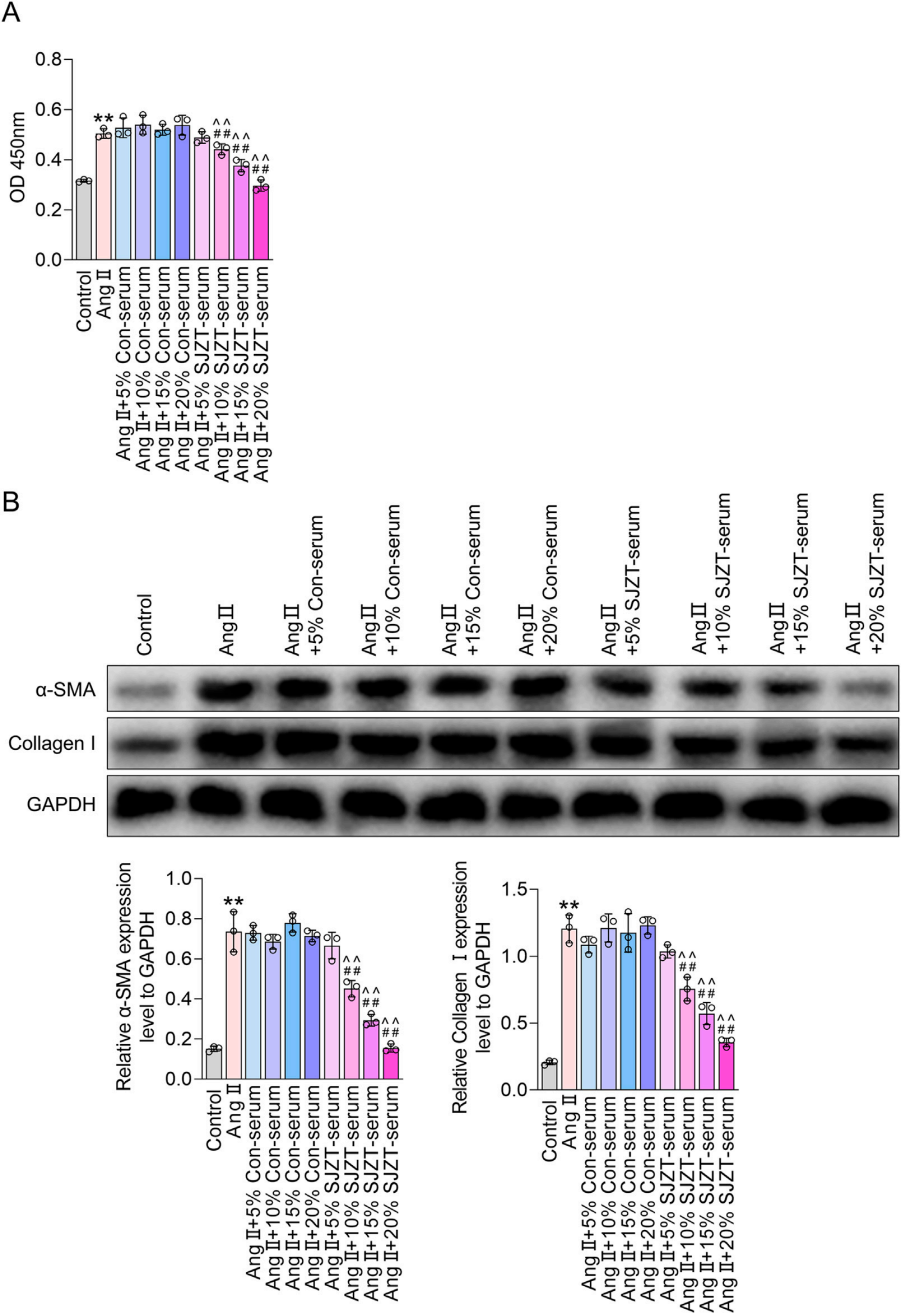
SJZT inhibits renal fibroblast activation. **(A,B)** Mouse kidney fibroblasts were treated with AngⅡ, or with 5%, 10%, 15%, and 20% serum, or with 5%, 10%, 15%, and 20% SJZT drug-containing serum. CCK-8 was used to detect cell viability **(A)**. Western blot was used to detected α-SMA and Collagen I **(B)**. **p < 0.01 VS Control, ##p < 0.01 VS Ang II, ^^p < 0.01 VS Ang II + Con-serum.

Fibroblast activation is the primary cytological basis for renal fibrosis, characterized by enhanced cell viability and increased expression of α-SMA and collagen I (An et al., 2022; Mack and Yanagita, 2015; Yan et al., 2016; Zhao et al., 2022). Therefore, we analyzed the effect of SJZT on fibroblast activation *in vitro*. The results demonstrated that Ang II treatment significantly elevated cell viability and upregulatedα-SMA and collagen I, indicating Ang II-induced renal fibroblast activation, whereas 10%, 15%, and 20% SJZT-containing serum effectively attenuated the Ang II-induced rise in these parameters, while 5% SJZT-containing serum had no effect (Figure 7). These findings suggest that SJZT mitigates renal fibroblast activation, and subsequent *in vitro* experiments employed 15% drug-containing serum.

### 3.9 SJZT inhibits renal fibroblasts activation by activating autophagy

**FIGURE 8:**
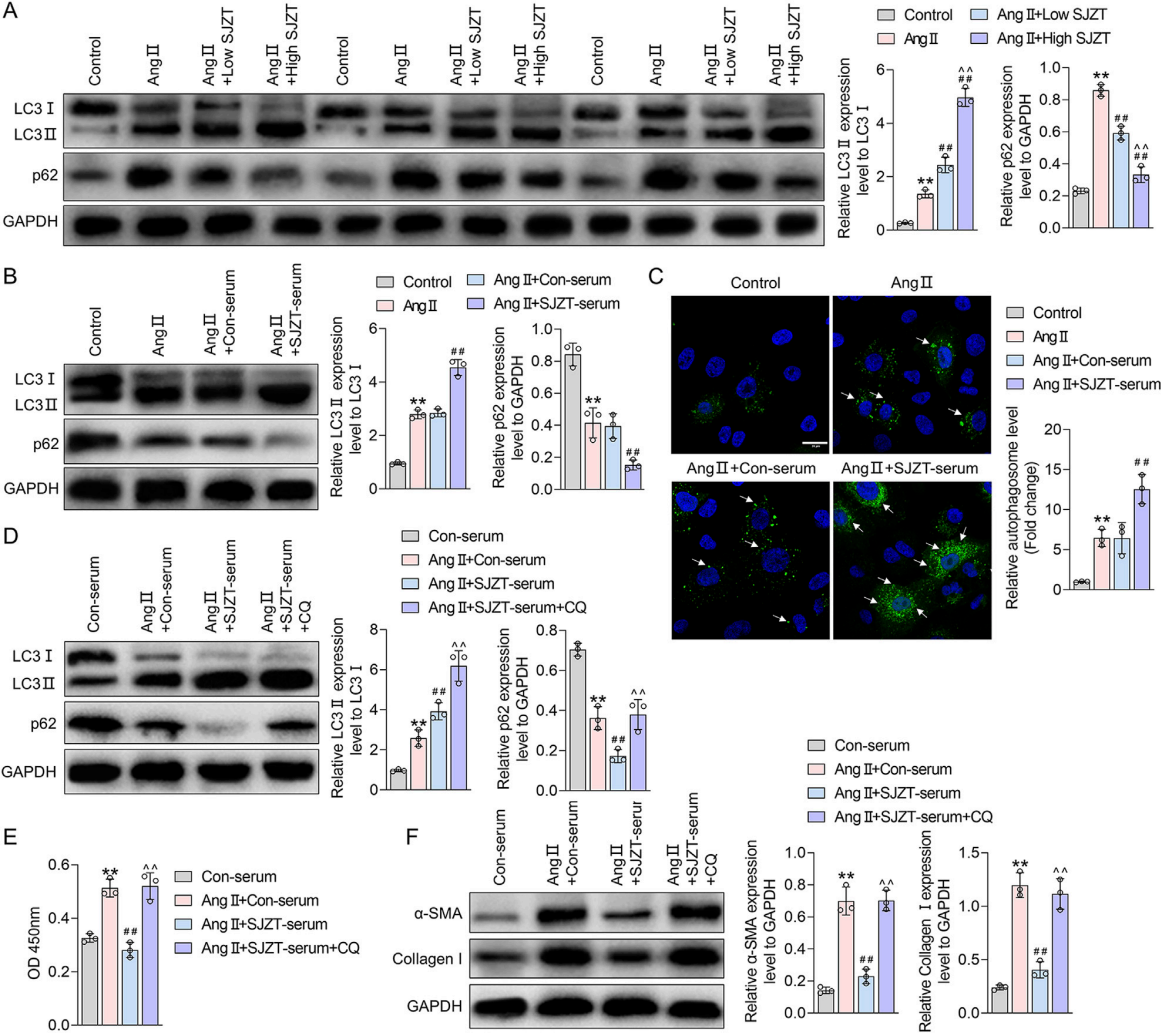
SJZT inhibits renal fibroblast activation by activating autophagy. Western blot analysis of LC3 and p62 levels in mouse kidney tissue **(A)** and renal fibroblasts **(B)**; **(C)** Ad-mCherry-GFP-LC3B virus was used to trace autophagy with a scale of 20 μm; **(D)** After protein extraction from renal fibroblasts, LC3 and p62 levels were detected by Western blot; **(E)** CCK-8 was used to detect the cell viability; **(F)** Western blot was used to detected α-SMA and Collagen I. **(A)** **p < 0.01 VS Control, ##p < 0.01 VS Ang II, ^^p < 0.01 VS Ang II + Low SJZT; **(B)** **p < 0.01 VS Control, ##p < 0.01 VS Ang II + Con-serum; **(D–F)** **p < 0.01 VS Con-serum, ##p < 0.01 VS Ang II + Con-serum, ^^p < 0.01 VS Ang II + SJZT-serum.

Renal fibrosis and fibroblast activation are linked to autophagy, therefore, we investigated whether SJZT modulates this process. In the Ang II-induced HN model, we found that the ratio of LC3II/LC3I and p62 levels were mildly elevated, whereas SJZT further increase the LC3II/LC3I ratio and dose-dependently attenuated the rise of p62, with greater efficacy at high dose (Figure 8A). *In vitro* renal interstitial fibroblasts, Ang II also induction mildly increased the LC3II/LC3I ratio but decreased p62 levels, SJZT also further elevated the LC3II/LC3I ratio and suppressed p62 levels (Figure 8B). Using Ad-mCherry-GFP-LC3B virus to monitor autophagy flux, we observed increased green fluorescence (indicating autophagosome accumulation) in the Ang II-induced group, which was further enhanced by SJZT (Figure 8C). These findings suggested that SJZT activates autophagy in renal interstitial fibroblasts.

To determine if SJZT inhibits renal interstitial fibroblasts activation via autophagy induction, we blocked autophagy with lysosomal inhibitor CQ, and found that CQ further elevated the LC3II/LC3I ratio and increased p62 (Figure 8D). Analyzed cell viability and protein level, we found that although SJZT suppressed cell viability and reduced α-SMA and collagen I levels, but CQ reversed these effects (Figures 8E,F). These findings suggested that SJZT inhibits renal fibroblast activation and myofibroblast formation by activating autophagy.

### 3.10 SJZT inhibits renal fibroblast activation by activating autophagy through PPARγ

**FIGURE 9:**
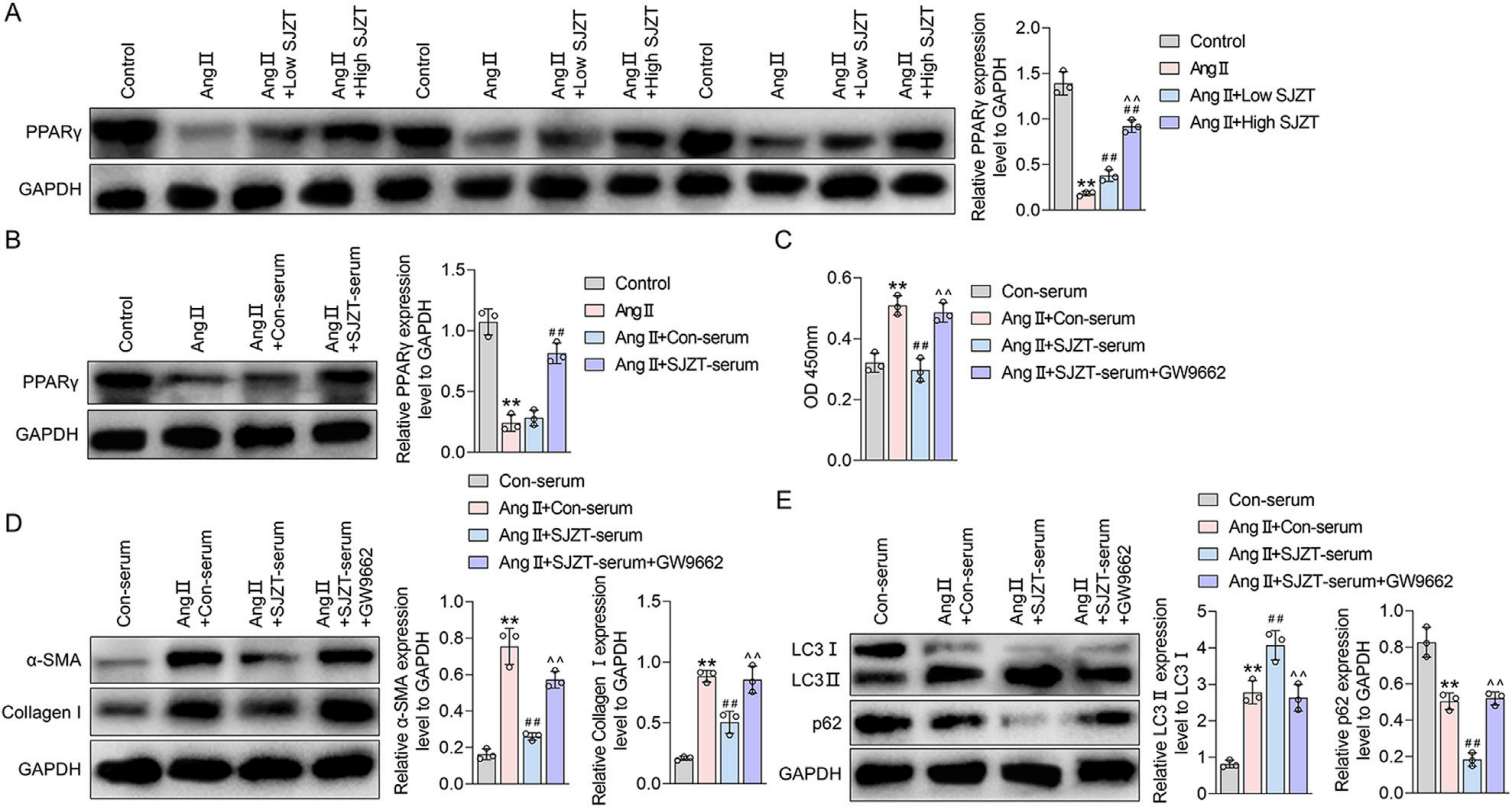
SJZT inhibits renal fibroblast activation by activating autophagy through PPARγ. Western blot was used to detected PPARγ in mouse kidney tissue **(A)** and renal fibroblasts **(B)**; **(C)** CCK-8 was used to detect the cell viability; **(D)** α-SMA and collagen I levels was detected by Western blot and quantitative analysis; **(E)** LC3 and p62 protein levels was detected by Western blot and quantitative analysis. **(A)** **p < 0.01 VS Control, ##p < 0.01 VS Ang II, ^^p < 0.01 VS Ang II + Low SJZT; **(B)** **p < 0.01 VS Control, ##p < 0.01 VS Ang II + Con-serum; **(C–E)** **p < 0.01 VS Con-serum, ##p < 0.01 VS Ang II + Con-serum, ^^p < 0.01 VS Ang II + SJZT-serum.

PPARγ signaling is a known inhibitor of renal fibrosis (Kokeny et al., 2021; Mu et al., 2022). Network pharmacological analysis suggests that SJZT active components target PPAR pathway (Sun et al., 2016). Specifically, we identified three SJZT active components (Licoisoflavone B, Glabrone, and Frutinone A) as direct PPARγ ligands. In the Ang II-induced HN model, PPARγ expression was significantly downregulated, whereas SJZT treatment dose-dependently restored PPARγ levels, with greater efficacy at high dose (Figure 9A). Similarly, *in vitro* renal interstitial fibroblasts, Ang II suppressed PPARγ, and SJZT reversed this suppression (Figure 9B). These finding suggested that SJZT increase PPARγ expression in renal interstitial fibroblasts.

Previous studies demonstrate that PPARγ promotes autophagy (Geng et al., 2024; Li Y. et al., 2024). To analysis whether SJZT inhibits renal fibroblast activation via PPARγ-mediated autophagy, we employed the PPARγ inhibitor GW9962. And found although SJZT significantly counteracted the Ang II-induced increase in fibroblast viability, whereas the effect abolished by GW9962 (Figure 9C). Western blotting further confirmed that SJZT attenuated Ang II-mediated upregulation of α-SMA and collagen I, while GW9962 reversed this effect (Figure 9D). Finally, SJZT enhanced autophagy, evidenced by elevated the LC3II/LC3I ratio and reduced p62 levels, and GW9962 blocked this effect (Figure 9E). These finding indicated that SJZT activates PPARγ-dependent autophagy to suppress renal fibroblasts activation.

## 4 Discussion

HN ranks as the second most common cause of ESRD, and its incidence continues to rise annually. Apart from dialysis and kidney transplantation, effective treatment options remain insufficient in clinical practice. Exploring novel therapeutic strategies and elucidating their underlying mechanisms are critical for HN management. In this study, we found that SJZT effectively inhibited renal fibrosis in an Ang II-induced HN mouse model. We further observed that SJZT suppressed renal fibroblast activation and myofibroblast formation, thereby reducing extracellular matrix (ECM) deposition. Mechanistically, the beneficial effect of SJZT were associated with the activation of PPARγ-mediated autophagy.

TCM is a feasible choice for treating HN because it is known for having few side effects and employs a holistic approach that targets multiple pathways and mechanisms. SJZT, a classical TCM formula, has been clinically utilized for treating diverse diseases, particularly gastrointestinal disorders, including dyspepsia, chronic gastritis, gastric cancer, irritable bowel syndrome, colorectal cancer, and ulcerative colitis. Its therapeutic effects are mediated through modulating gut microbiota homeostasis, attenuating inflammation, promoting mucosal repair, and enhancing immune regulation (Shang et al., 2023; Yang et al., 2025). After oral administration of SJZT, multiple bioactive components are detectable in plasma, including saponins, flavonoids, and terpenoids, exhibiting distinct ADME profiles. Although individual components exhibit low absorption efficiency, combinatorial formulation enhances the ADME of specific constituents. Specifically, the four-herb synergy increases bioavailability of primary components from *P. ginseng C.A.Mey.* and *Atractylodes Macrocephala Koidz*, with *G. uralensis Fisch* markedly improving ginsenoside bioavailability, while polysaccharides facilitate intestinal metabolism of ginsenosides and recirculation of their aglycone metabolites (Dong et al., 2023; Qu et al., 2018). It demonstrates the synergistic effect of the herb active components. The active components of SJZT have been confirmed by previous studies to ameliorate hypertensive kidney damage (Wang et al., 2019), and SJZT can suppress inflammation, renal fibrosis, and improve renal function in CKD (Yu et al., 2024). Therefore, SJZT shows therapeutic potential for HN. Current studies indicate that anti-fibrotic agents/herbs can mitigate renal damage through multi-target mechanisms, including fibrosis inhibition, antioxidant effects, and anti-inflammatory. However, these drugs carry risks of adverse reactions, including gastrointestinal discomfort and drug interactions, and their long-term safety and efficacy require further validation (Azizah et al., 2021; Dong et al., 2021). In contrast, SJZT, with about one thousand years of clinical application, has demonstrated a well-established safety profile. However, no study has systematically reported the active components, targets, and mechanisms of SJZT in treating HN.

Owing to the multi-component and multi-target of TCM formulas, conventional pharmacological approaches are often inadequate to systematically elucidate their pharmacological mechanisms. Network pharmacology, a research method combining computer technology and systems biology, explore the intricate relationships between TCM formulas and diseases, and it is significant for revealing their pharmacological mechanism (Singh et al., 2025). Molecular docking, a powerful technique for predicting ligand-target binding pattern and affinity, is crucial for elucidating these interactions (Bisht et al., 2024). Therefore, network pharmacology and molecular docking are instrumental in deciphering the complex mechanisms of TCM formulas in treating diseases, as well as identifying key active components and targets. In this study, we established a network pharmacology-based diagram of the SJZT active components and HN targets, encompassing 87 active components and 26 intersection targets. Among these, the top 10 core active components, including quercetin, DFV, Glyasperin F, Gancaonin A, arachidonate, Glabrone, Frutinone A, Licoisoflavone B, Shinpterocarpin, and kaempferol, were identified as potential core active components of SJZT in treating HN. Notably, quercetin inhibits renal fibrosis by blocking tubular epithelial cell ferroptosis and epithelial-mesenchymal transition (Gao et al., 2025; Lu et al., 2018; Wang et al., 2023), while kaempferol acts via the Hedgehog and ROS-ASK1-MAPK pathway (Guan et al., 2023; Sharma et al., 2020; Zhang et al., 2024). Functional enrichment and pathway analyses revealed significant enrichment in endothelial cell proliferation, regulation of blood pressure, reactive oxygen species metabolic process, prostaglandin receptor activity, G protein-coupled receptor binding, RAS, and MAPK signaling pathway, all implicated in renal fibrosis (Li, J. et al., 2024; Li X. et al., 2024; Livingston et al., 2024; Pohl and Schiessl, 2023). Through STING and Cytoscape analyses, PPARγ, TNF, CRP, ACE, HIF-1α, NF-κB, AGTR1, MPO, caspase 3 and F3 emerged as key targets of SJZT in treating HN, with molecular docking confirming strong binding of Licoisoflavone B, Glabrone, and Frutinone A to PPARγ. Collectively, SJZT likely treats HN via a multi-targets, multi-process, and multi-pathway mechanism.

At the animal levels, it was the first time found that SJZT could effectively alleviate renal fibrosis in HN mouse model. Given that fibrosis is a key pathological feature of HN, with renal interstitial fibroblasts-to-myofibroblasts conversion as its cellular basis, we further demonstrated SJZT inhibits this process. These results preliminarily confirmed that SJZT efficacy against HN *in vivo and vitro.* Mechanistically, SJZT activated PPARγ-mediated autophagy, a pathway supported by network pharmacology and molecular docking. Other studies also have demonstrated associations between the active components of SJZT and PPAR pathway (Sun et al., 2016). Experimental data further confirm the regulation effect of SJZT on PPARγ. Notably, PPAR activation exerts anti-fibrosis effects in HN, mediated through multifactorial mechanism, including anti-inflammation, anti-fibrosis, RAS suppression, vascular protection, and anti-apoptosis (Duan et al., 2023; Li et al., 2023). In addition, PPARγ also involves regulating autophagy (Manzeger et al., 2023). Autophagy, a lysosomal degradation process, modulates hypertension-related complications (Eisenberg et al., 2017; Forte et al., 2020; McCarthy et al., 2019) and renal fibrosis (Guo et al., 2019; Wu et al., 2023; Zhang and Liu, 2025). Currently, PPARγ is recognized as a therapeutic target for HN. PPARγ agonists, such as pioglitazone and rosiglitazone, have demonstrated renoprotective and antihypertensive effects in renal diseases (Ma et al., 2020). However, existing evidence primarily derived from animal studies, with limited clinical data (Gao and Gu, 2022; Kokeny et al., 2021). Additionally, synthetic PPARγ agonists exhibit certain side effects, the new generation of selective PPARγ modulators and natural agonists have emerged as a key research focus due to their reduced side-effect (Jia et al., 2014; Sharma and Patial, 2022). TCM, as a natural product, offers advantages such as minimal adverse effects and high safety, rendering it more clinically valuable. Notably, SJZT can activate PPARγ, suggesting greater potential for HN treatment compared to synthetic PPARγ agonists. Finally, this study has limitations: Firstly, validation was restricted to PPARγ and autophagy, future work must confirm other targets and pathways predicted by network pharmacology. Secondly, *in vitro* cell experiment relied solely to murine renal fibroblasts, human-derived renal fibroblasts should be employed to improve clinical relevance.

In summary, this study demonstrated that SJZT inhibits HN progression through multi-component, multi-target, and multi-pathway. Mechanistically, SJZT upregulated PPARγ expression, thereby activating autophagy, suppressing fibroblasts-myofibroblasts transition, and reducing Collagen I accumulation, ultimately mitigating renal fibrosis. These findings provide experimental evidence for SJZT clinical application in HN treatment.

## Data Availability

The original contributions presented in the study are included in the article/Supplementary Material, further inquiries can be directed to the corresponding author.
